# Diversity of *Anaplasma* and *Ehrlichia/Neoehrlichia* Agents in Terrestrial Wild Carnivores Worldwide: Implications for Human and Domestic Animal Health and Wildlife Conservation

**DOI:** 10.3389/fvets.2018.00293

**Published:** 2018-11-23

**Authors:** Marcos Rogério André

**Affiliations:** Laboratório de Imunoparasitologia, Departamento de Patologia Veterinária, Faculdade de Ciências Agrárias e Veterinárias, Universidade Estadual Paulista (FCAV/UNESP), Jaboticabal, Brazil

**Keywords:** *Anaplasma*, *Ehrlichia*, “*Candidatus* Neoehrlichia sp.”, carnivora, genetic diversity, ticks

## Abstract

Recently, the incidence and awareness of tick-borne diseases in humans and animals have increased due to several factors, which in association favor the chances of contact among wild animals and their ectoparasites, domestic animals and humans. Wild and domestic carnivores are considered the primary source of tick-borne zoonotic agents to humans. Among emergent tick-borne pathogens, agents belonging to family Anaplasmataceae (Order Rickettsiales) agents stand out due their worldwide distribution and zoonotic potential. In this review we aimed to review the genetic diversity of the tick-transmitted genera *Ehrlichia, Anaplasma and “Candidatus* Neoehrlichia sp.” in wild carnivores Caniformia (Canidae, Mustelidae and Ursidae) and Feliformia (Felidae, Hyanidae, Procyonidae and Viverridae) worldwide, discussing the implications for human and domestic animal health and wildlife conservation. Red foxes (*Vulpes vulpes*) have been identified as hosts for *Anaplasma* spp. (*A. phagocytophilum, Anaplasma ovis, A. platys*), *Ehrlichia canis* and “*Candidatus* Neoehrlichia sp.” (FU98 strain) and may contribute to the maintaenance of *A. phagocytophilum* in Europe. Raccoons (*Procyon lotor*) have been reported as hosts for *E. canis, A. bovis*, “*Candidatus* Neoehrlichia lotoris” and *A. phagocytophilum*, and play a role in the maintenance of *A. phagocytophilum* in the USA. Raccoon dogs (*Nyctereutes procyonoides*) may play a role as hosts for *A. bovis* and *A. phagocytophilum*. New *Ehrlichia* and/or *Anaplasma* genotypes circulate in wild canids and felids from South America and Africa. While *Ehrlichia* sp. closely related to *E. canis* has been reported in wild felids from Brazil and Japan, *Anaplasma* sp. closely related to *A. phagocytophilum* has been detected in wild felids from Brazil and Africa. Red foxes and mustelids (otters) are exposed to *E. canis* in countries located in the Mediaterranean basin, probably as a consequence of spillover from domestic dogs. Similarly, *E. canis* occurs in procyonids in North (raccoons in USA, Spain) and South (*Nasua nasua* in Brazil) Hemispheres, in areas where *E. canis* is frequent in dogs. While “*Candidatus* Neoehrlichia lotoris” seems to be a common and specific agent of raccoons in the USA, “*Candidatus* Neoehrlichia sp.” (FU98 strain) seems to show a broader range of hosts, since it has been detected in red fox, golden jackal (*Canis aureus*) and badger (*Meles meles*) in Europe so far. Brown (*Ursus arctos*) and black (*Ursus americanus*) bears seem to play a role as hosts for *A. phagocytophilum* in the North Hemisphere. *Anaplasma bovis* has been detected in wild Procyonidae, Canidae and Felidae in Asia and Brazil. In order to assess the real identity of the involved agents, future works should benefit from the application of MLST (Multi Locus Sequence Typing), WGS (Whole Genome Sequencing) and NGS (Next Generation Sequencing) technologies aiming at shedding some light on the role of wild carnivores in the epidemiology of Anaplasmataceae agents.

## Introduction

Recently, the incidence of tick-borne diseases in humans and animals have increased due to several factors, which in association favor the chances of contact among wild animals and associated ectoparasites, domestic animals and humans. The bi-directional flow of tick-borne parasites may occur from wildlife to domestic carnivores and vice-versa (1). Among the main factors associated with the emergence or re-emergence of vector-borne diseases, we can name: climate change, including global warming (for instance, shorter winters have been reported in continental areas of Europe, which impact the development and activity of ticks); “outdoor” activities, global traveling, urbanization, changes in land use, deforestation, habitat fragmentation, natural environment encroachment, which together predispose to a higher contact among wildlife, humans and domestic animals; the employment and easier access to molecular tools, favoring the diagnosis and identification of vector-borne agents; and the increase of awareness of tick-borne agents by veterinarians, physicians, scientists, and public health authorities. Regarding the latter, the veterinary practitioner play a central role and acts as a sentinel to alert epidemiologists, since they are the first one to notice the emergence of clinical cases (1–3).

Wild and domestic carnivores are considered the primary source of tick-borne zoonotic agents to humans. The dynamic of tick-borne agents transmission has been driven by different vertebrate host species living in sympatry. In this scenario, the overlapping of different species' ecological niches creates opportunities to parasites spread their geographical distribution, abundance and host range. As a consequence, several newly discovered arthropod-borne pathogens originated from wildlife has emerged, or reemerged [when a sudden peak of a certain disease occurs after a silent period (1)].

Among emergent tick-borne pathogens, agents belonging to family Anaplasmataceae (Order Rickettsiales) agents stand out due their worldwide distribution and zoonotic potential. Anaplasmataceae family comprises the genera *Ehrlichia, Anaplasma, Neorickettsia*, and *Wolbachia*. These agents are Gram-negative, small, most frequently pleomorphic, coccoid to ellipsoidal bacteria that reside within cytoplasmic vacuoles of the host cells (erythrocytes, reticuloendothelial cells, bone marrow-derived phagocytic cells, endothelial cells and cells of insect, helminth and arthropod reproductive tissues), either singly or, more frequently, in compact inclusions called morulae (4).

In this review we aimed to review the genetic diversity of the tick-transmitted genera *Ehrlichia, Anaplasma and “Candidatus* Neoehrlichia sp.” in terrrestrial wild carnivores worldwide, discussing the implications for human and domestic animal health and wildlife conservation. In the first section, we presented the molecular prevalence and diversity of Anaplasmataceae agents in wild carnivores Caniformia (Canidae, Mustelidae and Ursidae) and Feliformia (Felidae, Hyanidae, Procyonidae and Viverridae) around the world, including: (i) the previously recognized agents, namely *Anaplasma bovis* and *Anaplasma ovis, Anaplasma platys, Anaplasma phagocytophilum, Ehrlichia canis, Ehrlichia chaffeensis;* (ii) new *Candidatus* species [“*Candidatus* Neoerlichia lotoris” and “*Candidatus* Neoehrlichia sp.” (FU98) in the United States and Europe, respectively]; (iii) new genotypes of *Ehrlichia* and *Anaplasma* in Brazil and South Africa. Second, we presented previously reported findings related to the consequences associated to contact among wildlife-domestic animals-humans, such as: the effect of the infection by *Ehrlichia* and *Anaplasma* agents on wild carnivores health; the consequences of the contact between domestic dogs and wild canids on the exposure to *E. canis* in wild carnivores and vice-versa; the implications of *Ehrlichia* and *Anaplasma* agents infection in wild carnivores on human and domestic animals health; the role of coyotes (*Canis latrans*) in the epidemiological cycles of *Ehrlichia chaffeensis* in the USA; and the role of raccoons (*Procyon lotor*) in the epidemiological cycles of *Anaplasma phagocytophilum* in the USA and Europe. Finally, we presented the final remarks, highlighting the future directions of the research on the diversity of Anaplasmataceae agents in wild carnivores, emphasizing the need for the application of MLST (Multi Locus Sequence Typing), WGS (Whole Genome Sequencing) and NGS (Next Generation Sequencing) technologies in order to assess the real role of wild carnivores in the epidemiology of these group of α-proteobacteria worldwide.

Considering that interpretation of *Ehrlichia* spp. and *Anaplasma* spp.-reactive antibody titers detected in indirect fluorescent antibody (IFA) surveys among wild vertebrate hosts can be complicated by serological cross-reactions (5), we focused on studies that employed molecular techniques in order to confirm the identity of a certain Anaplasmataceae agent. Results of serological assays were only referenced when a lack of molecular studies precluded inferences related to the interaction between wild and domestic carnivores in the epidemiology of *Ehrlichia* and *Anaplasma* infections. Therefore, for the purpose of the present study, we search for the following index terms in Medline database: “wild carnivores,” “wild canids,” “wild felids,” “procyonids,” “mustelids,” “Hyaenidae,” “Ursidae,” “Viverridae” in association with “*Ehrlichia*,” “*Anaplasma*,” “*Neoehrlichia*,” and “Anaplasmataceae.”

## Molecular prevalence and diversity of tick-borne anaplasmataceae agents in terrestrial free-ranging and captive carnivores worldwide

### Anaplasma bovis

A molecular occurrence of 5.15% (36/699) for *A. bovis*, an Anaplasmataceae agent that parasitizes monocytes and macrophages (4), has been reported among raccoons in Hokkaido, Japan (6) (Table 1). Indeed, raccoons were imported as pets from North America to Japan due to the influence of the popular cartoon “Rascal Raccoon” on the television in 1977. However, when they eventually manifested their wild nature and became aggressive, these animals were intentionally released or run away from their homes, spreading through several areas of Japan (6). These medium-sized carnivores have been incriminated as potential reservoirs for *A. bovis* in Japan, playing a role in the maintenance of this agent in the environment. A statistical association between PCR-positivity for *A. bovis* and infestation by *Haemaphysalis* spp. ticks has been reported in raccoons from Japan (6). This agent was also recently detected in raccons dogs (*Nyctereutes procyonoides*) from Korea (2.1–6.6%) (14, 16). In South America, *A. bovis* 16S rDNA gene sequences (*rrs*) were detected in 4/31 (13%) coatis (*Nasua nasua*), a procyonid species in the Brazilian Pantanal (12). In Brazilian Pantanal, De Sousa et al. (12) detected *Anaplasma rrs* closely related to *A. bovis* in *Amblyomma* ticks collected from coatis (one *A. ovale* adult and *A. sculptum* nymphs).

**Table 1:** Molecular detection of *Anaplasma* spp. in free-ranging and captive terrestrial wild carnivores (Canidae, Mustelidae, Ursidae, Felidae, Hyaenidae, Procyonidae and Viverridae) around the world.

**Host**	**Technique (Target genes)**	**Result**	**Sample origin**	**References**
**Suborder Caniformia**			
**Family Canidae**
*Canis aureus* (golden jackal)	cPCR (*rrs*)	0/1 – *A. phagocytophilum*	Czech Republic (F)	(7)
	cPCR (*rrs/msp2*) qPCR/ RFLP *Anaplasma* spp. (*A. ovis, A. marginale, A. centrale, A. phagocytophilum*) (*msp4)*	2/216 (0.9%) – *A. phagocytophilum*	Serbia (F)	(8)
*Canis lupus* (gray wolf)	cPCR (*rrs*)	0/3	Brazil (C)	(9)
	qPCR: *Anaplasma* sp. (rrs) *A. marginale/A. ovis* (*msp-4*) *A. hagocytophilum* (*msp-2*) cPCR (*rrs*)	0/2	Spain (F)	(10)
*Canis mesomelas* (black backed jackal)	RLB (*rrs)* for *A. bovis, A. centrale, A. marginale, A. phagocytophilum, Anaplasma sp. Omatjenne, A. platys* cPCR (*rrs*)	82/142 (57.7%) *Anaplasma* sp. showing identity to A*. phagocytophilum* and *Anaplasma* sp. South African Dog	South Africa (F/C)	(11)
*Cerdocyon thous* (crab-eating fox)	cPCR (*rrs*)	0/39	Brazil (C)	(9)
	cPCR (*groEL*)	1/78 (1.2%) – *Anaplasma* sp. related to*A. bovis*	Brazil (F)	(12)
*Chrysocyon brachyurus* (maned wolf)	cPCR (*rrs*)	0/23	Brazil (C)	(9)
*Lycaon pictus* (African wild dog)	cPCR (*rrs*) RLB (*A. centrale A. marginale A. ovis, A. phagocytophilum 1, A. phagocytophilum 3, A. phagocytophilum 5, A. phagocytophilum* 7) (*rrs*)	0/301	South Africa (F)	(13)
*Nyctereutes procyonoides* (raccoon dog)	cPCR (*rrs*)	1/15 (6.6%) – *A. bovis*	Korea (F)	(14)
	cPCR *A. phagocytophilum* (*rrs*)	0/7	Czech Republic (F)	(7)
	cPCR *A. phagocytophilum* (*rrs*/*groEL*)	0/10	Poland (F)	(15)
	cPCR (*rrs*/*groEL/ankA/msp-2*)	2/193 (1%) – *A. phagocytophilum* 4/193 (2.1%) – *A. bovis*	Korea (F)	(16)
*Pseudalopex vetulus* (hoary fox)	cPCR (*rrs*)	0/8	Brazil (C)	(9)
*Speothos venaticus* (bush dog)	cPCR (*rrs*)	0/27	Brazil (C)	(9)
*Vulpes lagopus* (arctic foxes)	cPCR (*rrs*)	1/28 (3.6%) *Anaplasma* sp.	Canada (F)	(17)
*Vulpes vulpes* (red fox)	cPCR (*rrs*)	25/150 (16.6%) – *A. phagocytophilum*	Italy (F)	(18)
	cPCR (*msp4*)	1/13 (7.7%) – *A. ovis*	Italy (F)	(19)
	cPCR (*rrs*)	0/36 – *Anaplasma* sp.	Austria (F)	(20)
	qPCR (*msp-2*) cPCR (*rrs*/*ankA*)	10/122 (8.2%) – *A. phagocytophilum*	Germany (F)	(21)
	qPCR (*msp-2*) cPCR *(groEL)*	8/81 (9.9%) – *A. phagocytophilum*	The Netherlands (F)	(22)
	cPCR/qPCR (*rrs*)	10/69 (14.5%) – *A. platys*	Portugal (F)	(23)
	cPCR (*rrs, ankA*)	9/353 (2.55 %) – *A. phagocytophilum* 0/353 – *A. platys*	Romania (F)	(24)
	cPCR (*rrs*)	0/119 – *Anaplasma* sp.	Bosnia and Herzegovina (F)	(25)
	qPCR/cPCR (*rrs*)	51/415 (12.5%) – *A. phagocytophilum* 0/415 – *A. platys*	Hungary (F)	(26)
	qPCR for *Anaplasma* sp. (rrs) *A. marginale/A. ovis* (*msp-4*) *A. phagocytophilum* (*msp-2*) cPCR (*rrs*)	0/54	Spain (F)	(10)
	qPCR for *A. phagocytophilum/A. platys* (*rrs*)	4/162 (2.4%) – *A. phagocytophilum*	Switzerland (F)	(27)
	HRM for A*. phagocytophilum (rrs)*	0/195	Germany (F)	(28)
	cPCR (*rrs*)	0/12 – *Anaplasma* sp.	Spain (F)	(29)
	cPCR (*rrs*)	1/151 (0.65%) – *A. phagocytophilum*	Italy (F)	(30)
	cPCR (*rrs*)	1/114 (0.8%) – *A. phagocytophilum*	Czech Republic (F)	(7)
	qPCR – A*. phagocytophilum (msp-2)*	0/97	Italy (F)	(31)
	cPCR *(rrs/groEL)*	3/506 (0.6%) – *A. phagocytophilum*	Austria (F)	(32)
**Family Mustelidae**			
*Lutra lutra* (otter)	qPCR: *Anaplasma* sp. (rrs) *A. marginale/A. ovis* (*msp4*) *A. phagocytophilum* (*msp-2*) cPCR (*rrs*)	0/2	Spain (F)	(10)
	cPCR (*rrs*)	0/1	Czech Republic (F)	(7)
*Meles meles* (Eurasian badger)	qPCR *A. phagocytophilum* (*msp-2*)	0/40	The Netherlands (F)	(22)
	qPCR: *Anaplasma* sp. (rrs) *A. marginale/A. ovis* (*msp4*) *A. phagocytophilum* (*msp-2*) cPCR (*rrs*)	2/130 (1.5%) – *Anaplasma* sp.	Spain (F)	(10)
	cPCR (*rrs*)	0/3	Spain (F)	(29)
	cPCR (*rrs*)	0/3	Czech Republic (F)	(7)
*Martes foina* (stone marten)	qPCR: *Anaplasma* sp. (rrs) *A. marginale/A. ovis* (*msp4*) *A. phagocytophilum* (*msp-2*) cPCR (*rrs*)	0/22	Spain (F)	(10)
	cPCR (*rrs*)	0/10	Spain (F)	(29)
	cPCR (*rrs*)	0/4	Czech Republic (F)	(7)
	qPCR (*msp2*) – *A. phagocytophilum*	0/2	Hungary (F)	(33)
*Martes martes* (pine marten)	qPCR: *Anaplasma* sp. (rrs) *A. marginale/A. ovis* (*msp4*) *A. phagocytophilum* (*msp-2*) cPCR (*rrs*)	0/14	Spain (F)	(10)
*Mustela erminea* (stoat)	qPCR: *Anaplasma* sp. (rrs) *A. marginale/A. ovis* (*msp4*) *A. phagocytophilum* (*msp-2*) cPCR (*rrs*)	0/1	Spain (F)	(10)
*Mustela nivalis* (weasel)	qPCR: *Anaplasma* sp. (rrs) *A. marginale/A. ovis* (*msp4*) *A. phagocytophilum* (*msp-2*) cPCR (*rrs*)	0/6	Spain (F)	(10)
	qPCR (*msp2*) – *A. phagocytophilum*	0/2	Hungary (F)	(33)
*Mustela putorius* (polecat)	qPCR: *Anaplasma* sp. (rrs) *A. marginale/A. ovis* (*msp4*) *A. phagocytophilum* (*msp-2*) cPCR (*rrs*)	0/6	Spain (F)	(10)
	cPCR (*rrs*)	0/4	Czech Republic (F)	(7)
	cPCR (*rrs*)	0/3	Spain (F)	(29)
*Mustela sibirica* (weasel)	qPCR (*rrs*) cPCR (*rrs*)	$\frac{1}{2}*$	Korea (F)	(34)
	qPCR (*rrs*)	$\frac{1}{2}*$	Korea (F)	(35)
*Neovison vison* (American mink)	qPCR: *Anaplasma* sp. (rrs) *A. marginale/A. ovis* (*msp4*) *A. phagocytophilum* (*msp-2*) cPCR (*rrs*)	$\frac{1}{2}\;\left(50\%\right)$ – *Anaplasma* sp.	Spain (F)	(10)
	qPCR (*E. canis rrs*)	0/3	Spain (F)	(36)
**Family Ursidae**			
*Ursus americanus* (American black bear)	qPCR (*msp-2*) cPCR (*rrs*)	3/80 (4%) – *A. phagocytophilum*	USA (F)	(37)
	qPCR (*msp-2*)	30/288 (10%) – *A. phagocytophilum*	USA (F)	(38)
	cPCR (*rrs*)	2/68 (3%) – *A. phagocytophilum*	USA (F)	(39)
*Ursus arctos* (brown bear)	cPCR (*rrs*)	18/74 (24.3%) – *A. phagocytophilum*	Slovakia (F)	(40)
*Ursus arctos yesoensis* (Hokkaido brown bear)	cPCR *(rrs/gltA)* RLB (*rrs*)	2/13 (15%) – *Anaplasma* sp. (AP-sd)	Japan (F)	(41)
**Suborder Feliformia**			
**Family Felidae**			
*Acinonyx jubatus* (cheetah)	cPCR (*rrs*)	0/4	Zimbabwe (C)	(42)
*Caracal caracal* (caracal)	cPCR (*rrs*)	0/1	Brazil (C)	(9)
*Felis silvestris* (wildcat)	qPCR/cPCR *Anaplasma* sp. (*rrs*) *A. marginale/A. ovis* (*msp-4*) *A. phagocytophilum* (*msp-2*)	0/8	Spain (F)	(10)
*Felis lybica cafra* (South African wildcat)	cPCR (*rrs*)	1/6 (13%) – *A. phagocytophilum*	Zimbabwe (C)	(42)
*Herpailurus yagouaroundi* (jaguarondi)	cPCR (*rrs*)	0/19	Brazil (C)	(9)
*Leopardus pardalis* (ocelot)	cPCR (*rrs*)	0/15	Brazil (C)	(9)
	cPCR (*rrs*)	1/7 (14.2%) – *Anaplasma* sp. related to *A. bovis*	Brazil (F)	(12)
*Leopardus tigrinus (*little spotted cat)	cPCR (*rrs*)	4/25 (16%) –*Anaplasma* sp. related to *A. phagocytophilum* 4/25	Brazil (C)	(9)
*Leopardus wiedii* (margay)	cPCR (*rrs*)	0/2	Brazil (C)	(9)
*Leptailurus serval* (serval)	cPCR (*rrs*)	0/1	Brazil (C)	(9)
	cPCR (*rrs*)	$\frac{1}{2}\left(50\%\right)$ – *A. phagocytophilum*	Zimbabwe (C)	(42)
*Lynx lynx* (Eurasian lynx)	qPCR (*rrs*)	0/22 – *A. phagocytophilum*	Sweden (F)	(43)
*Oncifelis colocolo* (pampas cat)	cPCR (*rrs*)	0/1	Brazil (C)	(9)
*Panthera leo* (lion)	cPCR (*rrs*)	1/10 (10%) – *A. phagocytophilum*	Italy (C)	(44)
	cPCR (*rrs*)	0/12	Brazil (C)	(9)
	cPCR (*rrs*)	6/86 (7%) – *A. phagocytophilum*	Zimbabwe (C)	(42)
	cPCR/RLBH (*rrs*)	0/13	Botswana (F)	(45)
*Panthera onca* (jaguar)	cPCR (*rrs*)	0/6	Brazil (C)	(9)
*Puma concolor* (puma)	cPCR (*rrs*)	7/47 (16%) – *A. phagocytophilum*	USA (F)	(46)
	cPCR (*rrs*)	0/8	Brazil (C)	(9)
*Panthera tigris (tiger)*	cPCR (*rrs*)	0/8	Brazil (C)	(9)
*Prionailurus bengalensis euptilura (*Tsushima leopard cat)	cPCR (*rrs*)	2/13 (15%) – *A. bovis*	Japan (F)	(47)
*Prionailurus bengalensis euptilura (*Tsushima leopard cat)	cPCR (*rrs*)	2/29 (6.9%) – *A. bovis*	Korea (F)	(48)
*Prionailurus iriomotensis* (Iriomote cat)	cPCR (*rrs*)	0/33	Japan (F)	(47)
*Prionailurus viverrinus* (fishing cat)	cPCR (*rrs*)	0/1	Brazil (C)	(9)
**Family Hyaenidae**			
*Crocuta crocuta* (spotted hyaena)	RLB *Anaplasma* (*rrs*)	0/47	Namibia and South Africa (F/C)	(49)
*Parahyaena brunnea* (brown hyaena)	RLB *Anaplasma* (*rrs*)	0/15	Namibia and South Africa (F/C)	(49)
**Procyonidae**			
*Nasua nasua* (coati)	cPCR (*rrs*)	7/31 (22.5%) – *Anaplasma* sp. 4/7 (*Anaplasma* sp. closely related to *A. bovis*) 1/7 (*Anaplasma* sp. closely related to *A. phagocytophilum)*	Brazil (F)	(12)
*Procyon lotor* (raccoon)	cPCR (*rrs, groEL p44*)	14/57 (24.6%) – *A. phagocytophilum*	USA (F)	(50)
	cPCR (*rrs*)	0/187	Japan (F)	(51)
	cPCR (*rrs*) for *A. phagocytophilum*	0/169	USA (F)	(52)
	cPCR (*rrs*)	36/699 (5.15%) – *A. bovis*	Japan (F)	(6)
	cPCR (*rrs*)	0/15	Czech Republic (F)	(7)
	cPCR (*rrs*)	0/15	Czech Republic (F)	(7)
	cPCR (*rrs/groEL*)	1/78 (1.3%) – *A. phagocytophilum*	Poland (F)	(15)
	cPCR (*rrs/groEL*)	0/40	Germany (F)	(15)
**Family Viverrridae**			
*Genetta genetta* (common genet)	qPCR: *Anaplasma* sp. (rrs) *A. marginale/A. ovis* (*msp4*) *A. phagocytophilum* (*msp-2*) cPCR (*rrs*)	0/14	Spain (F)	(10)
	cPCR (*rrs*)	0/34	Spain (F)	(29)

C, captive; F, free-ranging; *Not sequenced.

Besides raccoons and raccons dogs, *A. bovis rrs* has also been detected in Tsushima leopard cats (*Prionailurus bengalensis euptilura*) sampled in Japan (15%) (47, 53) and Korea (6.9%) (48), and in *Haemaphysalis longicornis* ticks associated to these wild felids (47, 53). *A. bovis rrs* were also recently detected in ocelots (*Leopardus pardalis*) (14%) and crab-eating foxes (*Cerdocyon thous*) (1.3%) in the Brazilian Pantanal (12) (Table 1). This agent was also detected in a bush-dog (*Speothos venaticus*) maintained in captivity in a Brazilian zoo based on a PCR assay targeting *groEL* gene (9) (Table 1). In Thailand, *Anaplasma rrs* closely related to *A. bovis* was detected in three *Haemaphysalis lagrangei* ticks collected from a specimen of Malayan sun bear (*Helarctos malayanus*) (54).

### Anaplasma ovis

*Anaplasma ovis* was molecularly detected (based on *rrs* and *msp4* genes) in foxes (3.3%; 1/13) from Palermo and Ragusa provinces of Sicily, Italy. Out of 110 fleas collected from foxes, *Anaplasma* sp. was molecularly detected in 30% of them. Interesting, while one *Xenopsylla cheopis* flea showed copositivity for *A. ovis* (*msp4*) and *A. phagocytophilum (msp5)*, another one, from the same species, showed copositivity for *A. ovis (msp4)* and *A. marginale (msp4)*. These intriguing findings could be explained considering the fact that foxes in Sicily, Italy, are frequently found surrounding sheep farms. According to the authors, fleas may have acquired the found *Anaplasma* species from sheep, which served as prey for foxes. Although these findings do not incriminate fleas as vectors for *Anaplasma*, they suggest that siphonapterans may maintain these organisms (19).

### Anaplasma platys

*Anaplasma platys*, an Anaplasmataceae agent that parasitizes platelets (4), has been molecularly detected in a moderate rate (14.5%; 10/69) in foxes from Portugal (Table 1). This moderate proportion of positive foxes both in northern/central and southern Portugal suggests the existence of a sylvatic cycle of *A. platys* in this country, driven by the homogeneous distribution of this agent in the tick vectors. Foxes are incriminated as possible reservoirs for this agent for domestic dogs in Portugal. Indeed, the infection with *A. platys* seems to be more prevalent than that observed for *E. canis* in red fox populations in Portugal (23). Molecular evidence of the occurrence *A. platys* in humans has been reported in Venezuela (55) and in the USA (56), suggesting the possible zoonotic potential of this agent.

### Anaplasma phagocytophilum

*Anaplasma phagocytophilum*, a zoonotic agent that parasitizes neutrophils, is transmitted by ticks belonging to the *Ixodes persulcatus* complex, which are mainly found in the Northern hemisphere. This agent is mainly transmitted by *Ixodes persulcatus* in Asia and *Ixodes ricinus* in Europe, although *Ixodes trianguliceps* may also play an important role in the transmission of *A. phagocytophilum* among rodents. While *I. scapularis* is the vector of *A. phagocytophilum* in the eastern USA, *I. pacificus* is the main vector of this agent in the western USA [reviewed by (57)]. In addition to *I. pacificus*, the nidicolous tick species *I. angustus, I. ochotonae, I. spinipalpis*, and *I. woodi* may act as vectors for *A. phagocytophilum* in California, USA (58). This agent is responsible for causing the human granulocytic anaplasmosis (HGA) in northern hemisphere, equine and canine granulocytic anaplasmosis in the USA, and tick-borne fever in cattle and sheep in Europe [reviewed by (57)].

Since transstadial transmission of *A. phagocytophilum* in its vector ticks is well known, but transovarial transmission has not been demonstrated so far, vertebrate reservoir hosts are responsible for the maintenance of this agent in the environment (57).

Among wild canids, *A. phagocytophilum* has been molecularly detected in gray foxes (*Urocyon cinereoargenteus*) (9%) from northern California, USA (59), red foxes from Italy (0.5–16.6%) (18, 30), Germany (8.2%) (21), the Netherlands (9.9%) (22), Romania (2.55%) (24), Hungary (12.5%) (26), Switzerland (2.4%) (27), Czech Republic (0.8%) (7), Austria (0.6%) (32), raccoon dogs from Germany (23%) (21), golden jackals (*Canis aureus*) from Serbia (0.9%) (8), and raccons dogs (*Nyctereutes procyonoides*) from Korea (1%) (16). In Africa, *Anaplasma* sp. *rrs* closely related to *A. phagocytophilum* was detected in black backed jackals in South Africa (57.7%) (11) (Table 1). Recently, *A. phagocytophilum rrs* was detected in *Ixodes ricinus* collected from two foxes in Romania (60).

Among wild felids, *A. phagocytophilum rrs* has been molecularly detected in free-ranging mountain lions (*Puma concolor*) from California, USA (16%) (46), captive lions in Italy (10%) (44), captive little spotted cats (16%) in Brazil (9), and captive lions (7%), Southern Africa wild cats (13%), and servals (50%) (*Leptailurus serval*) in Zimbabwe (42) (Table 1).

Among procyonids, *A. phagocytophilum rrs* have been molecularly detected in raccoons (24.6%) from Connecticut, USA (50) and Poland (1.3%) (15), and coatis (3.2%) from Pantanal, Brazil (12) (Table 1). In Brazilian Pantanal, an *Anaplasma rrs* closely related to *A. phagocytophilum* was detected in *A. sculptum* nymphs collected from one coati (12).

Finally, *A. phagocytophilum* (*rrs* or *msp-2*) have been molecularly detected in free-ranging American black bears (*Ursus americanus*) (3–10%) in the USA (37–39) and free-ranging brown bears (*Ursus arctos*) in Slovakia (24.3%) (40) was reported in Slovakia (40) (Table 1).

Although *A. phagocytophilum rrs* have been detected in little spotted cats and coatis in Brazil (9), these animals were negative in qPCR assays targeting specific *msp-2* of *A. phagocytophilum* and conventional PCR directed to *groEL* gene, indicating that an *Anaplasma* genotype closely related to *A. phagocytophilum* circulate among wild carnivores in South America (Table 1). In Africa, although *A. phagocytophilum rrs* were detected in black backed jackals by reverse line blotting (RLB) (11), and lions, Southern African wild cats and servals by conventional PCR assays (Table 1), additional molecular characterization based on other genes was not performed (42). Therefore, the real identity of these *Anaplasma* genotypes circulating in wild carnivores in southern hemisphere, where recognized vectors of *A. phagocytophilum* don't occur, should be evaluated with caution. Future works aiming at isolating these new genotypes in order to carry out more accurate molecular characterization is much needed.

While serological and molecular prevalence rates for *A. phagocytophilum* of 89.5 and 24.6% have been reported among raccoons from Connecticut, USA (50), all 169 raccoons sampled in peridomestic areas in the states of Florida and Georgia showed to be negative in PCR assays for *A. phagocytophilum* (52). In another study, only one out of 156 raccoons from five populations sampled in the states of Georgia and Florida showed to be seropositive to *A. phagocytophilum* (61). Raccoons showed to be susceptible to experimental infection with a human-originated *A. phagocytophilum* strain (52). In Japan, one out 187 feral raccoons (0.5%) sampled in Kanagawa Prefecture, Japan, showed to be seropositive to *A. phagocytophilum* (51). Recently, *A. phagocytophilum* was detected in a raccoon (1.2%) from northwestern Poland (15). The *groEL* sequence analysis showed that the found *A. phagocytophilum* belongs to the European zoonotic ecotype I previously reported by Jahfari et al. (22) and Hildebrand et al. (15).

### Other *Anaplasma* genotypes

Non-characterized *Anaplasma groEL* sequences were detected in *Amblyomma* ticks from crab-eating foxes (*A. sculptum* adults and nymphs, *A. parvum* adult, *A. ovale* adult and *Amblyomma* larvae) and coatis (*A. sculptum* numphs and *A. ovale* adult) in Brazilian Pantanal (12). In Japan, *Anaplasma* sp. (AP-sd), previously reported in ticks, cattle, sika deer (*Cervus nippon yesoensis*) and rodents, was detected in wild Hokkaido brown bears (*Ursus arctos yesoensis*) (15%) (41).

### Ehrlichia canis

*Ehrlichia canis*, the agent of canine monocytic ehrlichiosis, infects monocytes and macrophages of domestic dogs and wild carnivores (62). *Rhipicephalus sanguineus* sensu lato (s.l.) (63) and *Dermacentor variabilis* (64) are the recognized vectors for *E. canis*. Although the brown dog tick *Rhipicephalus sanguineus* s.l. has been considered the primary vector of *E. canis* (65) showed, using experimental trials, that while the “tropical lineage” of *R. sanguineus* (populations from the state of São Paulo) showed vectorial competence for *E. canis*, the “temperate lineage” of this tick species (populations from Argentina, Uruguay, and southern Brazil) was not able to transmit this *Ehrlichia* species. In addition to *R. sanguineus* s.l. and *Dermacentor variabilis* (62), *Dermacentor marginatus* and *Ixodes canisuga* have been suggested as possible vectors of *E. canis* (66).

Molecular evidence of the occurrence *E. canis* in humans has been reported in Venezuela (67, 68) and Costa Rica (69), suggesting the zoonotic potential of this agent. Based on the amino acid tandem repeat sequence of the TRP36 protein, a novel genotype of *E. canis* was described in blood donors from Costa Rica, grouping within a single clade closely related to the Brazilian genogroup of *E. canis* detected in dogs (69).

Among wild canids, *E. canis rrs* has been detected in bush dogs (*Speothos venaticus*) (11.1%) and crab-eating foxes (*Cerdocyon thous*) (2.6–10.2%) from Brazil (9, 12), artic foxes (*Vulpes lagopus*) from Canada (3.6%) (17), red foxes (*Vulpes vulpes*) from Italy (31–52%) (19, 70, 30), Portugal (2.29%) (23) and Spain (16.6%) (29), gray wolves (*Canis lupus*) from Italy (50%) (70). An *E. canis dsb* sequence has been detected in one crab-eating fox maintained in captivity in a Brazilian zoo (9) (Table 2). In Brazilian Pantanal, (12) detected *Ehrlichia rrs* in *Amblyomma* ticks collected from crab-eating foxes (*A. parvum* adults, *A. sculptum* adults and nymphs, and *Amblyomma* larvae) and coatis (*A. sculptum* adults and nymphs and *Amblyomma* larvae). The *Ehrlichia rrs* detected in one *A. sculptum* adult and nymph, one *A. parvum* and one *Amblyomma* larvae pool were closely related to *E. canis* (12). In the USA, specific antibodies to *E. canis* were detected in 18% (9/50) coyotes sampled in Texas and Oklahoma, USA, using a p16 peptide-based microtiter plate ELISA (79).

**Table 2:** Molecular detection of *Ehrlichia* spp. in free-ranging and captive terrestrial wild carnivores (Canidae, Mustelidae, Ursidae, Felidae, Hyaenidae, Procyonidae, and Viverridae) around the world.

**Host**	**Technique (Target genes)**	**Result**	**Sample origin**	**References**
**Suborder Caniformia**			
**Family Canidae**			
*Canis aureus* (golden jackal)	cPCR (*rrs*/*groEL*)	0/1 – “*Candidatus* Neoehrlichia sp.” (FU98)	Czech Republic (F)	(7)
*Canis lupus* (gray wolf)	cPCR (*rrs*)	1/3 (33.3%) – *Ehrlichia* sp. related to *E. chaffeensis*	Brazil (C)	(9)
*Canis lupus* (gray wolf)	cPCR for *Ehrlichia* sp. (*rrs*)	0/2	Spain (F)	(10)
*Canis mesomelas* (black backed jackal)	RLB (*rrs*) for *E. canis, E. chaffeensis, E. ruminantium*, cPCR (*rrs*)	0/142	South Africa (F/C)	(11)
*Cerdocyon thous* (crab-eating fox)	cPCR (*rrs/dsb*)	4/39 (*rrs*: 10.2%); 1/39 (*dsb*: 2.5%) *Ehrlichia* sp. related to *E. canis* 4/39 (10.2%) – *Ehrlichia* sp. related to *E. chaffeensis*	Brazil (C)	(9)
	cPCR (*rrs*/*dsb)*	6/58 (10.3%) – *Ehrlichia* sp. related to *E. ruminantium*	Brazil (F)	(71)
	cPCR (*rrs*)	2/78 (7.6%) – *Ehrlichia* sp. related to *E. canis*	Brazil (F)	(12)
*Chrysocyon brachyurus* (maned wolf)	cPCR (*rrs*)	0/23	Brazil (C)	(9)
*Lycaon pictus* (African wild dog)	cPCR (*rrs*) RLB for *Ehrlichia* spp. (*Ehrlichia/Anaplasma* catch-all, *E. canis/E. ovina E. chaffeensis*) *E. ruminantium Ehrlichia* sp. (Omatjenne)	0/301	South Africa (F)	(13)
	cPCR *E. canis/E. ewingii* (*rrs*)	0/11	Zambia (F)	(72)
*Nyctereutes procyonoides* (raccoon dog)	cPCR (*rrs*)	0/15 – *Ehrlichia* sp.	Korea (F)	(14)
	cPCR (*rrs*/*groEL*)	0/7 – “*Candidatus* Neoehrlichia sp.” (FU98)	Czech Republic (F)	(7)
	cPCR (*rrs*/*groEL*)	3/10 (30%) – “*Candidatus* Neoehrlichia sp.” (FU98)	Poland (F)	(15)
*Pseudalopex vetulus* (hoary fox)	cPCR (*rrs*)	0/8	Brazil (C)	(9)
*Speothos venaticus* (bush dog)	cPCR (*rrs*)	3/27 (11.1%) – *E. canis*	Brazil (C)	(9)
*Vulpes lagopus* (arctic foxes)	cPCR (*rrs*)	1/28 (3.6%) – *E. canis*	Canada (F)	(17)
*Vulpes vulpes* (red fox)	cPCR (*rrs*)	4/13 (31%) – *E. canis*	Italy (F)	(19)
	cPCR (*rrs*)	0/36 – *E. canis*	Austria (F)	(20)
	cPCR/qPCR (*rrs*)	2/69 (2.9%) – *E. canis*	Portugal (F)	(23)
	cPCR (*rrs*)	0/353 – *E. canis*	Romania (F)	(24)
	cPCR (*rrs /groESL*)	1/164 (0.6%) – “*Candidatus* Neoehrlichia sp.” (FU98)	Austria (F)	(25)
	cPCR (*rrs*)	0/119 – *Ehrlichia* sp.	Bosnia and Herzegovina (F)	(25)
	qPCR/cPCR (*rrs*)	0/415 – *E. canis*	Hungary (F)	(26)
	cPCR for *Ehrlichia* sp. (*rrs*)	0/54	Spain (F)	(10)
	qPCR multiplex for “*Ca*. Neoehrlichia mikurensis,” “*Ca*. Neoehrlichia”, and Anaplasmataceae (*rrs*) qPCR for *E. canis* (*rrs*)	0/162	Switzerland (F)	(27)
	HRM for *E. canis* and “*Candidatus* Neoehrlichia mikurensis” (*rrs*)	0/195	Germany (F)	(28)
	cPCR (*rrs*)	2/12 (16.6%) – *E. canis*	Spain (F)	(29)
	cPCR (*rrs*)	55/105 (52%) – *E. canis*	Italy (F)	(70)
	cPCR (*rrs*)	68/151 (44.44%) – *E. canis*	Italy (F)	(30)
	cPCR (*rrs*/*groEL*)	1/114 (0.8%) -“*Candidatus* Neoehrlichia sp.” (FU98)	Czech Republic (F)	(7)
	qPCR (*rrs*-*E. canis*)	0/3	Spain (F)	(36)
	qPCR – “*Candidatus* Neoehrlichia mikurensis”(*groEL*)	0/97	Italy (F)	(31)
	cPCR *(rrs)*	2/506 (0.6%) “*Candidatus* Neoehrlichia sp.”(FU98)	Austria (F)	(32)
**Family Mustelidae**			
*Lutra lutra* (otter)	cPCR (*rrs*)	0/2	Spain (F)	(10)
	cPCR (*rrs*)	0/1	Czech Republic (F)	(7)
	qPCR/cPCR (*E. canis rrs*)	3/6 (50%)	Italy (F)	(73)
*Martes foina* (stone marten)	cPCR (*rrs*)	0/22	Spain (F)	(10)
	cPCR (*rrs*)	0/10	Spain (F)	(29)
	cPCR (*rrs*)	0/4	Czech Republic (F)	(7)
*Meles meles* (Eurasian badger)	cPCR (*rrs*)	1/130 (0.7%) *Ehrlichia* sp. related to *E. chaffeensis*	Spain (F)	(10)
	cPCR (*rrs*)	0/3	Czech Republic (F)	(7)
*Mustela erminea* (stoat)	cPCR (*rrs*)	0/1	Spain (F)	(10)
*Mustela nivalis* (weasel)	cPCR (*rrs*)	0/6	Spain (F)	(10)
*Mustela putorius* (polecat)	cPCR (*rrs*)	0/6	Spain (F)	(10)
	cPCR (*rrs*)	0/4	Czech Republic (F)	(7)
*Mustela sibirica* (weasel)	qPCR (*rrs*) cPCR (*rrs/nadA*)	$\frac{1}{2}*$	Korea (F)	(34)
*Neovison vison* (American mink)	cPCR (*rrs*)	0/2	Spain (F)	(10)
	qPCR (*E. canis rrs*)	0/3	Spain (F)	(36)
**Family Ursidae**			
*Ursus americanus* (American black bear)	cPCR *(rrs/gltA)*	0/49	USA (F)	(74)
**Suborder Feliformia**			
*Acinonyx jubatus* (cheetah)	cPCR (*rrs*)	0/4	Zimbabwe (C)	(42)
*Caracal caracal* (caracal)	cPCR (*rrs*)	0/1	Brazil (C)	(9)
*Felis silvestris* (wildcat)	cPCR (*rrs*)	0/8	Spain (F)	(10)
*Felis lybica cafra* (South African wildcat)	cPCR (*rrs*)	0/1	Zimbabwe (C)	(42)
*Herpailurus yagouaroundi* (jaguarondi)	cPCR (*rrs, omp1*)	1/6 (16.6%) – *Ehrlichia* sp. related to *E. canis*	Brazil (C)	(75)
*Herpailurus yagouaroundi* (jaguarondi)	cPCR (*rrs*)	2/19 (10.5%) – *Ehrlichia* sp. related to *E. canis*1/19 (5.3%) – *Ehrlichia* sp. related to *E. chaffeensis*	Brazil (C)	(9)
*Leopardus pardalis* (ocelot)	cPCR (*rrs, omp1*)	5/29 (17.2%) – *Ehrlichia* sp. related to *E. canis*	Brazil (C)	(75)
	cPCR (*rrs*)	2/15 (13.3%) – *Ehrlichia* sp. related to *E. canis* 2/15 (13.3%) – *Ehrlichia* sp. related to *E. chaffeensis*	Brazil (C)	(9)
	cPCR (*rrs*)	0/7	Brazil (F)	(12)
*Leopardus tigrinus* (little spotted cat)	cPCR (*rrs*)	2/14 (14.3%) – *Ehrlichia* sp. related to *E. canis*	Brazil (C)	(75)
	cPCR (*rrs*)	2/25 (8%) – *Ehrlichia* sp. related to *E. canis* 3/25 (12%) – *Ehrlichia* sp. related to *E. chaffeensis*	Brazil (C)	(9)
*Leopardus wiedii* (margay)	cPCR (*rrs, omp1*)	0/2	Brazil (C)	(75)
	cPCR (*rrs*)	0/2	Brazil (C)	(9)
*Leptailurus serval* (serval)	cPCR (*rrs*)	0/1	Brazil (C)	(9)
	cPCR (*rrs*)	0/2	Zimbabwe (C)	(42)
*Oncifelis colocolo* (pampas cat)	cPCR (*rrs, omp1*)	0/3	Brazil (C)	(75)
	cPCR (*rrs*)	0/1	Brazil (C)	(9)
*Panthera leo* (lion)	cPCR (*rrs*)	2/12 (16.6%) – *Ehrlichia* sp. related to *E. canis*1/12 (8.3%) – *Ehrlichia* sp. related to *E. chaffeensis*	Brazil (C)	(9)
	cPCR (*rrs*)	1/86 (1%) – *E. canis*	Zimbabwe (C)	(42)
	cPCR/RLBH (*rrs*)	0/13	Botswana (F)	(45)
	cPCR (*rrs*)	0/24	Zambia (F)	(72)
*Panthera onca* (jaguar)	cPCR (*rrs, omp1*)	2/9 (22.2%) – *Ehrlichia* sp. related to *E. canis*	Brazil (C)	(75)
	cPCR (*rrs, dsb*)	2/10 (20%) – *Ehrlichia* sp. related to *E. ruminantium*	Brazil (F)	(76)
	cPCR (*rrs*)	0/6	Brazil (C)	(9)
*Puma concolor* (puma)	cPCR (*rrs, omp1*)	1/9 (11.1%)	Brazil (C)	(75)
	cPCR (*rrs*)	2/8 (25%) – *Ehrlichia* sp. related to *E. canis* 2/8 (25%) – *Ehrlichia* sp. related to *E. chaffeensis*	Brazil (C)	(9)
*Panthera tigris (tiger)*	cPCR (*rrs*)	2/8 (25%) – *Ehrlichia* sp. related to *E. chaffeensis*	Brazil (C)	(9)
*Prionailurus bengalensis euptilura* (Tsushima leopard cat)	cPCR (*rrs*)	1/8 (8%) – *E. canis*	Japan (F)	(47)
*Prionailurus iriomotensis* (Iriomote cat)	cPCR (*rrs*)	4/33 (12%) – *E. canis*	Japan (F)	(47)
*Prionailurus viverrinus* (fishing cat)	cPCR (*rrs*)	0/1	Brazil (C)	(9)
**Family Hyaenidae**			
*Crocuta crocuta* (spotted hyaena)	cPCR *E. canis/E. ewingii* (*rrs*)	0/19	Zambia (F)	(72)
	RLB *Ehrlichia* (*rrs*)	0/47	Namibia and South Africa (F/C)	(49)
*Parahyaena brunnea* (brown hyaena)	RLB *Ehrlichia* (*rrs*)	0/15	Namibia and South Africa (F/C)	(49)
**Family Procyonidae**			
*Nasua narica* (white-nose coati)	cPCR (*rrs/dsb*)	0/20 – *Ehrlichia* sp. 0/20 – “*Candidatus* Neoehrlichia lotoris”	Costa Rica (F)	(77)
*Nasua nasua* (coati)	cPCR (*rrs*)	1/31 (3.8%) – *Ehrlichia* sp. related to *E. canis*	Brazil (F)	(12)
*Procyon lotor* (raccoon)	cPCR (*rrs*)	1/60 (1.7%) – *E. canis* 32/60 (53.3%) – “*Candidatus* Neoehrlichia lotoris”	USA (F)	(78)
	cPCR (*rrs*)	0/187	Japan (F)	(51)
	cPCR (*rrs*) for *E. canis, E. ewingii, E. chaffeensis*	0/169	USA (F)	(52)
	cPCR (*rrs*)	131/197 (67%) – “*Candidatus* Neoehrlichia lotoris”	USA (F)	(61)
	cPCR (*rrs*)	0/15	Czech Republic (F)	(7)
	qPCR (*E. canis rrs*)	5/194 (2.57%)	Spain (F)	(36)
	cPCR *Neoehrlich*ia sp. (*rrs/groEL*)	0/78	Poland (F)	(15)
	cPCR *Neoehrlich*ia sp. (*rrs/groEL*)	0/40	Germany (F)	(15)
**Family Viverridae**			
*Genetta genetta* (common genet)	cPCR (*rrs*)	0/14	Spain (F)	(10)
	cPCR (*rrs*)	0/34	Spain (F)	(29)

C, captive; F, free-ranging; *Not sequenced.

Among wild felids, *E. canis rrs* has been detected in ocelots (*Leopardus pardalis*) (13.3–17.2%), jaguarondis (*Herpailurus yagouaroundi*) (10.5–16.6%), little spotted cats (*Leopardus tigrinus*) (8.0–14.3%), pumas (*Puma concolor*) (1.1–25%), jaguars (*Panthera onca*) (22.2%), lion (*Panthera leo*) (16.6%) maintained in captivity in zoos in Brazil (75, 9), free-ranging Iriomote cats (12%) (*Prionailurus iriomotensis*) and Tsushima leopard cats (*Prionailurus bengalensis euptilura*) (8%) in Japan (47), and lions (1%) maintained in captivity in Zimbabwe (42) (Table 2). Among procyonids and mustelids, *E. canis rrs* has been detected in raccoons (*Procyon lotor*) (1.7%) from the USA (78) and Spain (2.6%) (36), coatis (3.2%) from Brazil (12), and Eurasian otters (50%) from Italy (73) (Table 2). Although *E. canis rrs* has been detected in several wild captive felid species in Brazil (75), phylogenetic analysis based on *omp-1* gene positioned *Ehrlichia* sp. *omp-1* sequences obtained from three ocelots and one jaguar in a separated clade from *E. canis* and *E. chaffeensis* sequences, suggesting the occurrence of a new *Ehrlichia* species in wild felids in Brazil (75). Similarly, even though *E. canis rrs* was detected in crab-eating foxes and coatis in Brazilian Pantanal, these samples showed negative results in specific qPCR assays targeting *E. canis*-dsb gene (12). Considering that the majority of the *E. canis* sequences obtained from wild carnivores worldwide are based only on short *rrs*, future studies regarding the genetic diversity of *Ehrlichia* spp. in wild animals should focus on different target genes other than *rrs*, in order to assess the real identity of these newly reported Anaplasmataceae genotypes as discussed in section Final remarks and future directions.

Red and gray foxes (80) showed to be susceptible to experimental infection by *E. canis* from dog-infected blood. Additionally, the gray fox was able of providing an infectious blood meal for *R. sanguineus* larvae (80). Millán et al. (29) observed that foxes inhabiting natural areas in periurban Barcelona, Spain, showed a high frequency of infection by *E. canis* when compared to dogs from surrounding residential areas. Likewise, *E. canis* seems to be frequent among wild canids (foxes and gray wolves) in Italy, suggesting that a sylvatic life cycle of this pathogen may occur in that country (70). On the other hand, *E. canis* (2.9%) showed to be less prevalent than *A. platys* (14.5%) in red foxes from Portugal. These findings may be related to a heterogeneous distribution of the agents within the vector populations in Europe (23). Recently, *R. sanguineus* s.l. ticks collected from vegetation (questing) or from domestic and wild animals (including three red foxes) from 18 administrative regions of mainland Portugal, showed to belong to the “temperate lineage” and were negative in PCR assays for *E. canis* (81). In southern Brazil, where a “temperate lineage” of *R. sanguineus* is present, sampled dogs showed positive results in PCR assays for *A. platys* but not for *E. canis* (82). Further studies are needed in order to investigate the role of other tick species in *E. canis* transmission cycles among dogs and foxes in Portugal.

In Palermo and Ragusa provinces of Sicily, Italy, prevalence rates for *E. canis* (*rrs*) of 31% (4/13) and 3% (3/110) were reported among foxes and associated fleas. While 2 positive fleas were collected from foxes that were also positive for *E. canis*, a third positive flea was collected from a fox that was negative for this pathogen. Although fleas have not been recognized as vectors for Anaplasmataceae agents so far, the detection of *E. canis* in the third flea may suggest that this insect might be involved in the maintenance of *E. canis* (19). Similarly, *Ehrlichia* sp. *rrs*, closely related to *E. canis*, was detected in two *Amblyomma sculptum* ticks, non-recognized vectors for *E. canis*, collected from *Ehrlichia* sp.-PCR positive wild crab-eating foxes in Brazilian Pantanal (12). In this case, PCR-positivity in ticks could be related to the remnant of infected host blood meal (12).

In North America, the detection of *E. canis* and *Anaplasma* sp. in arctic foxes in Canada provides evidence that these tick-borne pathogens, which has not been frequently associated with the arctic ecosystem, may circulate, even at low levels, in that sampled *Vulpes lagopus* population. Even though arctic foxes can occasionally interact with foxes and domestic dogs, these findings may reflect the consequences of direct or indirect human activity on natural environments, such as the global warming that may contribute to the spread of ticks and associated pathogens to this unique ecosystem. Therefore, arctic foxes may act as sentinels for the assessment of climate change on the emergence and eco-epidemiology of tick-borne zoonotic agents (17).

In the African continent, canine monocytic ehrlichiosis was incriminated as cause of death of several wild dogs (*Lycaon pictus*) in Kruger National Park, South Africa, although the confirmation of the aetiological agent was not performed at that time (83). When a black-backed jackal (*Canis mesomelas*) was experimentally infected with *E. canis*, it did not develop clinical signs, but remain a chronic carrier up to 112 days (83). Later, a case of fatal ehrlichiosis in a black-backed jackal following the exposure to ticks was reported in a kennel at the Onderstepoort Veterinary Research Institute, South Africa (84). Wild dogs and black-backed jackals showed to be susceptible to experimental infection with *E. canis*. Even though all experimental infected wild canids showed the presence of morulae in stained blood smears, while the first showed clinical and hematological abnormalities compatible with canine monocytic ehrlichiosis (depression, anorexia, pancytopaenia), the latter showed to be asymptomatic. Wild dogs appeared to be more resistant than domestic dogs, with a longer incubation period, despite higher levels of bacteremia. Moreover, clinical and haemotological abnormalities showed to be less severe and intensive treatment was not required. Besides, the disease was then successfully transmitted from experimentally infected black-backed jackals to domestic dogs (85), confirming the findings described by Neitz and Thomas (83). Then, *E. canis* was detected in eight out of 15 free-living jackals (*Canis mesomelas*) in Kenya, using a modified cell culture test (86). Therefore, jackals have been incriminated as reservoirs for *E. canis* (83, 85). The presence of antibodies to *E. canis* was detected in 34% (14/55) of free-ranging black backed jackals sampled in Kenya (87). Despite these findings, *E. canis* has not been molecularly detected in spotted (*Crocuta crocuta*) and brown (*Parahyaena brunnea*) hyenas, wild dogs, and black backed jackals in Zambia, Namibia and South Africa so far (13, 72, 49, 11). Interestingly, *E. canis rrs* was detected in 1 out of 86 captive lions in Zimbabwe (42).

Although *E. canis* has been molecularly detected in free-ranging raccoons from the USA (1.7%) (78) and Spain (2.6%) (36), Yabsley et al. (52) showed that *Procyon lotor* was not susceptible to experimental infection with *E. canis*. In the USA, although *D. variabilis*, a recognized vector for *E. canis* (64), has been found parasitizing dogs and medium-sized wild mammals, such as raccoons (52, 61, 78), allowing for *E. canis* inter-species transmission in suburban areas, raccoons seem to play a limited role as a vertebrate reservoir for this agent in the USA (78). Among 60 raccoons sampled in the state of Georgia, molecular and serological evidence of exposure to *E. canis* of 1.7 ands 21.7% were reported (78). Later, all 169 raccoons sampled in peridomestic areas from counties located in the states of Florida and Georgia, USA, showed to be negative in PCR assays for *E. canis*, despite seropositivity rates of 17.4 and 6.9% in the aforementioned states, respectively (52). In Japan, all 187 raccoons sampled in Kanagawa Prefecture showed to be PCR-negative for *E. canis*, despite the serological evidence of exposure to this agent in one animal (51) (Table 2). According to Criado-Fornelio et al. (36), *E. canis* may be tick-transmitted between domestic dogs and wild carnivores, including raccoons, and vice-versa in central Spain. In Brazil, among 31 coatis sampled in the Pantanal wetland, 3.2% showed to be seropositive to *E. canis* and presented *E. canis rrs* in blood sample (12). Therefore, the real role of procyonids in the epidemiological cycles of *E. canis* in nature should be further investigated.

### Ehrlichia chaffeensis

In the USA, *Ehrlichia chaffeensis*, the causative agent of human monocytotropic ehrlichiosis (HME), is maintained in a complex cycle involving the white-tailed deer (*Odocoileus virginianus*) and the lone star tick (*Amblyomma americanum*), which play a role as its primary reservoir and vector, respectively [reviewed by (88)]. Even though wild tailed deer act as the main host for *E. chaffeensis*, serological and molecular evidence of infection by this agent has been reported in wild carnivores.

Among wild carnivores, *E. chaffeensis rrs* has been detected in free-ranging coyotes (*Canis latrans*) (71%) from the USA, crab-eating foxes (10.2%), and gray wolves (33.3%), little spotted cats (12%), ocelots (13.3%), pumas (25%), jaguarondis (5.3%), lion (8.3%), and tigers (25%) maintained in captivity in Brazilian zoos (9). A genotype showing 97.3% identity to *E. chaffeensis* was detected in badgers (*Meles meles*) (1.5%) and in one (1/2) American mink (*Neovison vison*) from Spain (10) (Table 2). Although *Ehrlichia rrs* closely related to *E. chaffeensis* was detected in wild captive carnivores in Brazil (9), additional specific qPCR assays targeting *vlpt* gene of *E. chaffeensis* yielded negative results, emphasizing the possible occurrence of a genotype closely related to, albeit distinct from, *E. chaffeensis* in other regions of the world outside the USA. Considering that additional molecular characterization targeting other genes of these genotypes detected in mustelids from Spain was not performed, the real identity of these agents remains unknown. Considering that badgers' food habit can range from roots and fruits, earthworms, insects to small terrestrial vertebrates (including hedgehogs) and their cadavers, these wild animals may get infected with pathogens circulating in vertebrate tissues. Moreover, since badger populations can reach high numbers in urban environments, the role of these wild carnivores in the epidemiology of tick-borne diseases should be further addressed (10).

Although serological evidence of exposure to *E. chaffeensis* associated to parasitism by ticks including *A. americanum*, the recognized arthropod vector for this agent, has been reported among raccoons from Georgia, USA (28.7–38.3%) (52, 78) and Florida (34.8%) (52), when these wild carnivores were experimentally infected with *E. chaffeensis*, the infection course showed to be transient (52). While *E. chaffeensis* was isolated in cell culture from one experimentally infected raccoon, molecular and serological evidence of infection was reported in two out of the five experimentally infected raccoons (52). Indeed, all 169 raccoons sampled in peridomestic areas from counties located in the states of Florida and Georgia showed to be negative in PCR assays for *E. chaffeensis* (52) (Table 2). Therefore, they seem not to play a role as important reservoirs in the enzootic cycles of *E. chaffeensis* due to the transient or lacking rickettsemia observed during the experimentally course of infection (52).

Red foxes (*Vulpes vulpes*), but not gray foxes (*Urocyon cinereoargenteus*), showed to be susceptible to experimental infection with *E. chaffeensis* based on isolation of the agent from blood, seroconversion, and positive PCR results from spleen and lymph nodes samples. However, neither morulae in stained blood smears nor clinical signs were observed in experimentally infected animals. Considering that red foxes are also susceptible to *E. canis* infection (80) and keeping in mind the occurrence of serological cross-reactions among *Ehrlichia* species in the IFA, antibodies to *E. chaffeensis* or other *Ehrlichia* species in foxes should be interpreted with caution.

*Ehrlichia chaffeensis vlpt* was detected in in 1 of 23 (4.3%) *A. americanum* collected from black bears (*Ursus americanus floridanus*) in Georgia, USA (89). Recently, *E. chaffeensis rrs* was detected in 2/46 (4%) adult *A. americanum* ticks removed from brown bears (*Ursus americanus*) in Oklahoma, southcentral USA. Despite the negative results in PCR assays for *Ehrlichia* spp., all sampled brown bears (*n* = 49) showed to be seropositive to *E. chaffeensis* by IFAT (74).

### Ehrlichia ewingii

Although *E. ewingii* DNA has not been detected in wild canids so far, specific antibodies to this agent were detected in 46% (23/50) coyotes sampled in Texas and Oklahoma, USA, using a p28 peptide-based microtiter plate ELISA (79). Indeed, this agent seems to be the most common *Ehrlichia* species in coyotes from areas where *A. americanum* is prevalent (79).

### Genotypes related to *Ehrlichia ruminantium*

A genotype (Strain Jaguar) related to, albeit distantly from, *E. ruminantium* was detected in jaguars (2/10; 20%) and in associated tick species (*A. triste, A. sculptum*, and *Amblyomma* sp.) sampled in Pantanal wetland, central-western Brazil, based on *rrs* and *dsb* sequences (76) (Table 2). Later, a similar genotype (fox-ES-1) was detected in crab-eating foxes (6/58; 10.3%) in southeastern Brazil (71) (Table 2). These two genotypes formed a sister group of the *E. ruminantium* group (71, 76).

### Other *Ehrlichia* genotypes

Recently, *Ehrlichia* sp. HF *rrs* has been detected in specimens of *Ixodes apronophorus* collected from dogs and foxes in Romania (90). This *Ehrlichia* strain has been already detected in *I. ricinus* ticks in France (91) and in dogs, rodents and *Ixodes ovatus* ticks in Japan (92–94). Further studies aiming at investigating the role of foxes as linking mammals carrying ticks from infected rodents in the wild to dogs in suburban areas are necessary in Europe. An *Ehrlichia* sp. *rrs* genotype closely related to *E. chaffeensis/E. muris* was recently detected in an *Ixodes ricinus* female tick collected from a fox in Romania (60).

### “*Candidatus* neoehrlichia lotoris” (CNL)

Based on a partial *rrs* gene fragment, an Anaplasmataceae agent closely related to “*Candidatus* Neoehrlichia mikurensis” was detected in 53% of tested raccoons (*Procyon lotor*) sampled in the Piedmont region of Georgia, USA (78). This agent was then isolated in ISE6 tick cell culture (95). Even though the culture was able to infect raccoons, detectable infection was not seen in laboratory mice, rats and rabbits (61). Yabsley et al. (96) showed, using phylogenetic analyses based on three target genes (*rrs, groEL*, and *gltA*), that this new agent (CNL) isolated from a raccoon in a tick cell line (95) was closely positioned to, but distinct from, TK4456^R^ and IS58 strains of “*Candidatus* Neoehrlichia mikurensis.” Differences in sequences of the three target genes were not found between the strain RAC413^R^ of CNL and samples obtained from naturally infected raccoons from three states in the USA. No serological cross-reactivity with *E. chaffeensis, E. canis, E. ewingii, A. marginale* and *A. phagocytophilum* antigens was noted in four raccoons experimentally infected with CNL (RAC413^R^ strain). According to the authors, the lack of cross-reactivity between CNL and other Anaplasmataceae agents might be due to a weak antibody response developed by raccons to CNL antigens.

The fact that neither CNL nor “*Candidatus* Neoehrlichia sp.” (FU98) (see below) have been confirmed in populations of raccoons in Europe (15) and Japan (6) support the hypothesis that the narrow specificity of CNL to native populations of raccoons in North American presumably relates to a vector (15).

### “*Candidatus* neoehrlichia sp.” (FU98)

A new agent, so called “*Candidatus* Neoehrlichia sp.” (FU98), was detected in a fox spleen sample (0.6%) from Austria. The obtained *rrs* and *groEL* sequences showed to be closely related to the raccoon associated “*Candidatus* Neoehrlichia lotoris” from North America but clearly distinct from the *Ixodes ricinus* transmitted zoonotic “*Candidatus* Neoehrlichia mikurensis” found in Eurasia (97). Later, this agent was also detected in foxes (0.8%) from Czech Republic (7), in an European badger (*Meles meles*) from Hungary (98) and, more recently, in foxes (0.4%) in Western Austria (32) and in raccoon dogs (30%) from Poland (15) (Table 2). More studies are necessary in order to isolate this new agent as well as investigate its geographic distribution and host range.

## Impact of anaplasmataceae agents on wild carnivores health and conservation

Although *Ehrlichia* and *Anaplasma* infections have been reported in wild carnivores worldwide, few clinical cases of erhlichiosis and anaplasmosis have been described so far in this group of mammals. For instance, an outbreak of canine monocytic ehrlichiosis with high mortality and associated to a high *R. sanguineus* s.l. infestation was reported among wolves, dogs and wolf–dog hybrids in a zoo in Florida, USA (99).

In experimental studies carried out in South Africa, *E. canis* was successfully transmitted from domestic dogs to African wild dogs (*Lycaon pictus*) and black-backed jackals (*Canis mesomelas*). While the latter showed no clinical signs of infection, wild dogs showed clinical (anorexia and depression) and hematological (anemia, leucopaenia and thrombocytopaenia) signs of canine monocytic ehrlichiosis. The success of experimental infection trails was confirmed by the presence of morulae in leucocytes from experimentally infected wild carnivores. Besides, blood samples from jackals showed to be infective to domestic dogs (85). Moreover, coyotes, gray and red foxes showed to be susceptible to experimental infection with *E. canis* (80, 100).

In Italy, foxes naturally infected by *A. phagocytophilum* presented nonspecific microscopic alterations, such as mild lymphoreticular hyperplasia of the splenic follicles primarily localized to cortical areas (18).

A male timber wolf (*Canis lupus occidentalis*) maintained in captivity in an outdoor enclosure in Austria and naturally infected by *A. phagocytophilum* showed clinical (tick infestation, anorexia, depression) and hematological (thrombocytopenia, lymphopenia, mild anemia) signs of canine granulocytic anaplasmosis (101).

Indeed, the real impact of these pathogens on wild carnivores health has been seldom investigated, mainly because these animals have been incriminated as potential reservoirs for these agents. As a consequence, little effort has been made in order to deeply investigate the clinical course of the diseases caused by *Ehrlichia* and *Anaplasma* agents in wild carnivores. However, such sort of studies is much needed before assuming the role of these mammals as reservoirs (29).

## Could the contact between domestic dogs and wild canids drive a higher exposure to *E. canis* in wild carnivores and vice-versa?

Susceptible animal population may suffer from generalist pathogens “spill over” from abundant domestic reservoir hosts. In this specific case, control strategies should be directed to the domestic reservoir hosts. Alternatively, when a certain pathogen is transmitted within a threatened population, the effort of control measures over the wild population will favor the health management of the susceptible population rather than taking actions on another sympatric host species (102). Aiming at investigating interspecific and intraspecific transmission routes of exposure of South African wild dogs to *E. canis* and other selected canine pathogens, (102) analyzed behavioral measures of opportunities for contact between domestic dogs and other wild dogs. As a result, wild dogs presenting higher contact with domestic dogs were at higher risk of exposure to *E. canis*. On the other hand, contact with other wild dogs did not increase their exposure to *E. canis*. Indeed, exposure to *E. canis* was associated with small instead of large pack size. According to the authors, the lack of evidence of higher risk of acquiring *E. canis* within large packs might be explained by the deleterious effect of canine monocytic ehrlichiosis in wild dog puppies, which will promote high mortality rates and, consequently, diminishes the pack size. Similarly, both the increase of wild dog density over time and inter-pack contact was not associated to higher *E. canis* exposure. Therefore, domestic dogs may play a role as reservoir hosts for *E. canis*, since higher exposure to this pathogen was observed when wild dogs were in contact with domestic dogs. Alternatively, *E. canis* might be maintained in low-density wild dog populations, without the involvement of another host species. Keeping in mind that *E. canis* infection does not require direct contact between hosts since it is transmitted by ticks, the contact with domestic dogs would drive an elevation of the prevalence of this agent among wild dog populations rather than being necessary for the pathogen persistence in wild dog packs (102).

Although not confirmed, a decline in wild dog populations in South Africa was suspected to being caused by ehrlichiosis (83). However, despite the report of *E. canis* detection in 8 of 15 free-living jackals (*Canis mesomelas*) in Kenya using a modified cell culture test (86), this agent has not been molecularly detected in wild canids in Africa so far (11, 13, 49, 72). In the presence of higher contact with domestic dogs, and consequently, higher exposure to *E. canis*, a weaknesses status of the wild dog population analyzed in the studied performed by (102) would be expected. In fact, the wild dog packs sampled in the abovementioned study was growing and healthy. According to the authors, the level of contact between wild dogs and domestic dogs might have contributed to the development of immunity in the studied wild dog packs, preventing canine monocytic ehrlichiosis outbreaks and mortality. On the other hand, habitat fragmentation might favor a more frequent contact between both populations and, as consequence, promote higher exposure to *E. canis* and mortality (102).

In another scenario, red foxes *(Vulpes vulpes)* are suspected to play a role as a reservoir in the epidemiology of canine ehrlichiosis in Europe (66). In Hungary, domestic dogs, red foxes and golden jackals (*Canis aureus*) share the same tick species. Due to the spread of its geographic occurrence, golden jackals may act as carriers of Mediterranean ticks toward north Europe. The detection of *E. canis* DNA in *I. canisuga* larvae collected from red foxes in Hungary may represent the missing link between the domestic and sylvatic cycles of *E. canis*, involving foxes as reservoirs and dogs as susceptible hosts (66).

In Israel, similar seroprevalence rates to *E. canis* were reported among free-ranging golden jacks (35.8–54-3%) and stray dogs (37.5%) (103–105). The high seropositivity rates to *E. canis* found among jackals may be due to the ubiquitous presence of *R. sanguineus* s.l. ticks in Israel. Besides, the frequent incursions of golden jackals into urban areas in order to find food leftovers, associated to the close phylogenetic relation between jackals and dogs, may facilitate the spread of some pathogens to domestic dogs (104, 105). Among sampled jackals, three (9.7%) showed thrombocytopenia and, of these, two were seropositive to *E. canis*. Besides, three out of jackals presenting low haematocrit were seropositive to the studied agent. These findings indicated that free-ranging jackals in Israel might act as subclinical carriers of the pathogen (105).

## Implications of anaplasmataceae infection in wild carnivores on human and domestic animals health

Since wild carnivores are free ranging, the odds for being exposed to arthropod-vectors carrying pathogens are expected to be higher than those found among humans and domestic dogs. Therefore, the exposure rate to Anaplasmataceae agents is expected to be higher in wild carnivores than for domestic dogs and cats and humans. In fact, wild sentinels are more likely to reflect changes in ecological patterns of disease occurrence when compared to humans and domestic animals. While the reporting of tick-borne diseases in humans depends mostly on the definition of case reports, the prevalence of such diseases in domestic dogs and cats is more prone to be affected by factors that may mask the real circulation of vector-borne agents, such as the regular application of ectoparasiticides, preventive use of endoparasiticides, vaccination and antibiotic therapy. Moreover, wild carnivore surveillance may better define areas at higher risk for exposure to tick-borne agents, mainly because its wide geographic living range. On the other hand, domestic dogs and cats living area are mostly restricted to areas surrounding the activity zones of their owners (106).

The understanding about the exposure dynamics to tick-borne agents in wild carnivores is a crucial step in order to conduct accurate surveillance programs aiming at defining areas at higher risk of exposure to vector-borne pathogens. For instance, Jara et al. (106) investigated the exposure of gray wolves (*Canis lupus*) from different age groups (puppies, yearlings and adults) to two important tick-borne pathogens for humans and domestic dogs, namely *A. phagocytophilum* and *E. canis*, respectively, in the state of Wisconsin, USA. According to the authors, wolf seroprevalence is higher in adults than puppies for both studied tick-borne pathogens. On the other hand, seroprevalence in yearlings is similar to that one found among adults. Considering that antibodies can last long periods of time since the first exposure, it is more likely that adults show a higher chance to be exposed to certain pathogen during their life span. Besides, keeping in mind that the process of acquisition of Ixodidae ticks is a passive phenomenon, i.e, ticks that will transmit pathogens are sedentary and can be found waiting for vertebrate hosts on grasses and bushes, for example, the exposure to these arthropod vectors are associated to the movement rates of wild carnivores. While puppies are more likely to stay near the den sites, yearlings and adults are more prone to explore wider geographic areas and, consequently, being exposed to tick infested areas (106). Although white-tailed deer (*Odocoileus virginianus*) and white footed mice (*Peromyscus leucopus*) act as the main hosts for the black-legged tick *Ixodes scapularis* (the vector for *A. phagocytophilum*) and the main reservoirs for *A. phagocytophilum* in the Midwestern and Northeastern USA, respectively [reviewed by (57)], gray wolves can also play a role as hosts for both tick vectors and *A. phagocytophilum* (106).

In order to deal with habitat fragmentation and degradation, several wild carnivore species have developed the ability to adapt themselves to periurban/urban environments. As a consequence, the chances for contact among wild carnivores, domestic animals and humans have risen, facilitating the transmission of vector-borne agents. Because of that, the role that carnivores may play in the epidemiology of tick-borne pathogens of public health and veterinary importance has been investigated worldwide (29).

Foxes are the most widespread and abundant wild carnivores in Europe (1, 24). The red foxes has adapted and become a successful species in the urban environment, mainly due to the availability of food and resting places, lacking of predators, and human tolerance (1). During their excursions into suburban and urban environments looking for food, these wild carnivores are responsible for several troubles, such as predation of chickens and rabbits, fossicking trashcans and damaging gardens (19). As a result, the red fox plays a role as a linking between wild and urban environments. Indeed, its huge population size and widespread abundance make this wild carnivore species an important reservoir for pathogens that infect vertebrates sharing the same areas, including humans (1). Similarly to the red fox, the golden jackal (*Canis aureus*) is a generalist predator that is expanding its geographical distribution, being adaptable to any sort of environment and showing impressive capacity of colonizing different habitats (1).

These wild carnivores may be infested with tick and flea species acquired from associated prey or from other animals sharing the same environment (19). Considering that foxes may carry Anaplasmataceae-infected ticks, they may act as infection source for both domestic dogs and humans (30). Their adaptation to urban environment and human presence make them an important key in the ecoepidemiology of VBP in synanthropic environments (19, 24). Keeping that in mind, carrier wild carnivores may represent a potential source of Anaplasmataceae infection for hunters, professionals working with wildlife (veterinarians, zoo keepers), people residing and working in rural settlements, and people living in urban areas invaded by these animals (30).

Gabriel et al. (59) listed gray foxes (*Urocyon cinereoargenteus*) as good sentinels for *A. phagocytophilum* infections in northwestern California. Gray fox populations can be found at high densities in areas where HGA cases are reported in the USA. Moreover, these wild carnivores can share areas with domestic animals and humans. In a surveillance study conducted in Hoopa Valley Indian Reservation in Humboldt County, northern California, USA, Gabriel et al. (59) observed that the seroprevalence (70%) to *A. phagocytophilum* was higher in backcountry foxes than in urban-zone foxes (39%), which, in turn, showed a similar seroprevalence to that one found among domestic dogs (31%). Therefore, periurban foxes could transport *A. phagocytophilum*-infected ticks from wild environments to urban areas, contributing to tick exposure to domesticated animals and humans. Despite the seroprevalence rates, only 9% out of the 70 fox samples were PCR-positive for *A. phagocytophilum*, including four (13%) of the 30 urban and two (6%) of the 34 backcountry foxes (59).

In the USA, 47 mountain lions (*Puma concolor*) were sampled in Sierra Nevada foothills, northern coast range, and Monterey County in California. Among them, 17 and 16% showed positive results in serology and PCR, respectively, to *A. phagocytophilum* (46). According to the authors, even though wild canids and mountain lions do not play a role as important reservoirs of *A. phagocytophilum*, due to infrequently found PCR positive results, these mammals may be competent sentinels for the detection of this agent. This epidemiological role as sentinel is favored by their relatively large home ranges, long life spans, and common exposure to tick-vectors (46, 59).

Wild carnivores inhabiting natural areas in periurban Barcelona showed to be infested and infected, respectively, by ticks and fleas and vector-borne pathogens that infect dogs and cats, albeit with a higher frequency of infestation/infection. The contact between wild carnivores and domestic dogs may occur during scent communication behavior or during prey sharing. When it comes to transmission of vector-borne pathogens, the lack of necessity for direct contact between infected carrier wild carnivores and domestic dogs draw a favorable scenario for this group of pathogens (29).

Based on *groEL* sequences, (22) grouped *A. phagocytophilum* isolates from Europe in four ecotypes. Although ecotype I showed the widest host range, birds and rodents isolates were not found circulating in this ecotype. Considering that the *A. phagocytophilum* sequences isolated from red foxes and humans were found in this ecotype highlight the fact that some genotypes in this cluster are zoonotic. According to the authors, the generalist feeding behavior of *I. ricinus* nymphs and adults, a recognized vector for *A. phagocytophilum* in Europe, may facilitate the spread of ecotype I among different vertebrate host species, including humans. In Germany, *A. phagocytophilum ankA* sequences obtained from raccoons and red foxes clustered in *ankA* gene cluster I (107), which contains *A. phagocytophilum* strains from humans, dogs, and horses (21). Recently, *A. phagocytophilum groEL* sequences detected in red foxes in Austria showed to belong to a G-variant previously detected in humans, and domestic and wild animals (32).

Although foxes may play a limited impact on the circulation of emerging zoonotic *A. phagocytophilum* due to low to moderate infection rates (ranging from 0.5 to 16.6%), these wild carnivores may still represent a potential source of human infections (21, 24, 32) and a risk for the urbanization of the *A. phagocytophilum* life cycle (26). Molecular phylogenetic assessments from previous studies conducted in Europe evidenced that foxes were infected with the same strains of *A. phagocytophilum* previously reported in human patients (21, 22, 32).

Even though wild carnivores have been incriminated as sentinels for Anaplasmataceae agents in natural environments, as well as in degraded and periurban areas (29, 31) emphasized that the molecular screening of TBPs in vector ticks represents a more efficient system than the screening of foxes as sentinel animals in the specific epidemiological context of northeastern Italy (Belluno Province). Despite the detection of *A. phagocytophilum* and “*Candidatus* Neoehrlichia mikurensis” in *Ixodes ricinus* nymphs and adults, all sampled foxes (*n* = 97) were negative to Anaplasmataceae agents. Recently, *A. phagocytophilum msp-2* sequence was detected in one (2.3%) out 43 *Ixodes ricinus* collected from 90 foxes in Slovakia (108). Besides, *A. phagocytophilum rrs* was detected in *I. ricinus* ticks collected from two foxes in Romania (60, 90).

A positive role of golden jackals (*Canis aureus*) in the ecosystems has been reported based on the services these animals provide by removing a substantial amount of animal waste through their diet. However, the recent detection of *A. phagocytophilum* (0.2%) in these animals in Serbia brought up the fact that these wild carnivores may play a role as potential carriers of vector-borne zoonotic pathogens (8). In Israel, a seroprevalence of 26% to *A. phagocytophilum* was detected among a population of 53 free-ranging golden jackals; 5.7% of which were only seropositive to *A. phagocytophilum*, without any seroreactivity to either *E. canis* or *E. chaffeensis* (104). Considering that jackals are scavengers and usually are seen invading urban areas and feeding on garbage, there is a real risk of these wild canids carry *A. phagocytophilum*-infected ticks to the urban area, favoring the transmission of this agent to domestic dogs and human beings (104).

Similarly, bears may share living areas with wild and domestic canids. Likewise wild canids and raccoons, black bears have adapted to living in proximity to humans, which can result in trash cans rummage, car strikes and higher probability of pathogens transmission. In fact, molecular prevalence rates for *A. phagocytophilum* ranging from 3 to 10% in black bears in the USA (37–39) to 24% in brown bears in Europe (40) have been reported. On the other hand, this agent was not detected in 86 ticks collected from 17 black bears in the state of Lousiania, USA (109). The low levels of infection in bears in the USA may indicate a spillover phenomenon for this pathogen (39). While a seroprevalence rate of 26% (54/210) to *A. phagocytophilum* was reported among a black bears population in California, USA, a higher seroprevalence rate (65.2%) was reported among brown bears in Slovenia, central Europe (110). These results emphasize the need of a better surveillance of black and brown bears as additional potential reservoirs for this zoonotic agent in the USA and Europe (38, 40).

### The role of coyotes and raccoons in the epidemiological cycles of *Ehrlichia chaffeensis* in the USA

In the USA, although white-tailed deer (*Odocoileus virginianus*) and *Amblyomma americanum* are incriminated as the main reservoir and vector hosts, respectively, for *E. chaffeensis*, coyotes (*Canis latrans*) can play a role as bridge mammals in spreading this tick-borne agent. This key position in the epidemiological cycle of human monocytic ehrlichiosis in the USA is due to their following biological features: they act as vertebrate hosts for all *A. americanum* stages, show a wider geographic home range (31 km) when compared to WTD (1.6 km), and are susceptible to *E. chaffeensis* infection. Therefore, these wild canids may contribute to the dispersion of *E. chaffeensis*-infected ticks in the environment, which may subsequently feed on domestic animals and humans (111).

Raccoons (*Procyon lotor*), besides being abundant, show a wide geographical range in the USA. Indeed, they can be found in distinct ecologic niches. Considering the high numbers of raccoons are usually found in urban and suburban areas, they can easily may be in contact with domestic animals and humans (52). Although raccoons have been found naturally infected by *E. canis* (78), they seemed not to be susceptible to experimental infection with *E. canis* and *E. ewingii* (52). On the other hand, these mammals were susceptive to *E. chaffeensis* in experimental trials. *E. chaffeensis-*experimentally infected raccoons showed a transient pattern of infection: only two of three exposed raccoons became infected. *A. americanum* nymphs did not acquire the pathogen when fed on a single infected raccoon (52).

### The role of raccoons in the epidemiological cycles of *Anaplasma phagocytophilum* in the USA and europe

Raccoons have been incriminated as an important reservoir for *A. phagocytophilum* in the eastern United States. A dual pattern of infection was observed when raccoons were experimentally infected by two different *A. phagocytophilum* strains: while a long-lasting infection (at least 76 days) was observed in raccoons infected by a human isolate of *A. phagocytophilum*, a transient infection was found in raccoons experimentally infected by a white-tailed deer isolate of *A. phagocytophilum*. Despite the source of *A. phagocytophilum* strains, both group of experimentally infected raccoons seroconverted (52).

Besides being usually exposed to *A. phagocytophilum* in the environment, raccoons are highly susceptible to and proved to be able to transmit the agent to competent ticks. In fact, when compared to white-footed mice (*Peromyscus leucopus*), which also acts as a remarkable reservoir host for *A. phagocytophilum* in the USA, raccoons play an important role in the amplification of *A. phagocytophilum* infection in engorged nymphs (50). A plethora of reasons supports the hypothesis that raccoons contribute to the maintenance of *A. phagocytophilum* in the environment (50):

1. Raccoons have been found more parasitized by *A. phagocytophilum*-infected ticks when compared to white-footed mice. This observation is mainly due to a higher infestation density in this procyonid species, which comprises medium-sized mammals, comparatively to mice that encompass small-sized mammal specimens.
2. Higher seropositivity rates to *A. phagocytophilum* were observed in raccoons when compared to those found among white-footed mice. This difference in *A. phagocytophilum* exposure rate can be mainly attributable to a higher infestation of *A. phagocytophilum*-infected ticks in raccoons. In the second, the higher replacement rate in the mouse population can also affect the rates of seropositivity among white-footed mice.
3. Higher chances (almost twice) of capturing *A. phagocytophilum*-bacteremic raccoons were observed in field works when compared to those found for white-footed mice. This finding is mainly due to higher exposure rates to the agent, and/or to higher levels of bacteremia in raccoons.
4. Raccoons seem to amplify the *A. phagocytophilum* infection in the tick population. The prevalence of infection in ticks feeding on raccoons was higher than in those fed on white-footed mice and in questing ticks collected at the same location.
5. *A. phagocytophilum* DNA was detected in nymphs fed as larvae under similar proportions of tested raccoons and white-footed mice.
6. Nymphal ticks that fed as larvae upon raccoons transmitted *A. phagocytophilum* to naive mice.

Raccoons, originally from North America, are considered invasive species in Europe. Their expansion and harm effect on native fauna have raised fervent discussions surrounding this issue, mainly because of the lack of natural enemies and their fast pacing growing, making their control virtually impossible. Although little is known about the role of raccoons in the *A. phagocytophilum*-epidemiological cycles in Europe, a low frequency (0.8%) of infection has been recently reported among a population of raccoons sampled in Hungary (15).

## Final remarks and future directions

As shown in this work, the majority of molecular studies performed around the world aiming at detecting and characterizing Anaplasmataceae agents in wild carnivores used *rrs* as the only target gene for both screening and/or molecular characterization. The referred gene has been extensively used for molecular investigations on Anaplasmataceae agents due to its high conservation. Whether, on one hand, it allows the detection of new genotypes in wildlife, on the other hand, the phylogenetic assessment based on short *rrs* gene fragments does not provide sufficient genetic discrimination to allow the identification of *Ehrlichia* and *Anaplasma* species (9, 12). Moreover, previously described PCR protocols used for amplification of target genes other than *rrs* have been proven unsuitable for amplification of variants of *Anaplasma* and *Ehrlichia* species infecting wild mammals in Brazil, as previously reported (9, 12, 112).

The low bacteremia level in non-reservoirs wild carnivore blood or spleen samples often results in variable amplification of different target genes, precluding accurate prevalence investigations and phylogenetic assessments (12). Moreover, the primer sequences designed for more variable genes can be too dissimilar to anneal properly in the genic regions from newly discovered genotypes (113). Regarding the screening of wild carnivores populations for Anaplasmataceae agents, sensitive and specific broad range quantitative real-time PCR assays are desirable, instead of species-specific qPCR assays for an individual *Ehrlichia* or *Anaplasma* species. Recently, a sensitive and specific duplex qPCR assay targeting *groEL* sequences of *Anaplasma* and *Ehrlichia* species was designed (112) and used in the molecular screening for this group of agents in wild carnivores in Brazil (12). The performance of such assay in catching different variants of *Ehrlichia* and *Anaplasma* in other geographical regions should be further addressed.

Attempts to molecularly characterize new Anaplasmataceae genotypes in wild carnivores based on more evolving target genes are imperative in order to assess the real identity of the new strains. For instance, while *rrs*-based phylogenetic inference grouped the *Ehrlichia* sequences found in wild felids in Brazil together with the *E. canis* clade, the analysis based on *omp-1* gene positioned the same samples in a different and unique clade, indicating the circulation of another species/genotype in these wild carnivores in South America (75).

In order to deal with low bacteremia in biological samples, the isolation of new *Ehrlichia* and *Anaplasma* strains in mammal or tick-derived cell lines is needed aiming at describing a new species. For instance, the ultrastructure and an accurate molecular characterization and phylogenetic positioning of “*Candidatus* Neoerlichia lotoris” from raccoons from the USA (based on *rrs, groEL* and *gltA* genes) (95, 96) and *Ehrlichia minasensis* nov. sp. (*rrs, groEL, dsb, gltA* and *trp36* genes) (114) from a *Rhipicephalus microplus* tick from Brazil was only possible after the isolation of these agents in ISE6 and IDE8 cell lines, respectively. As far as we are concerned, no *Ehrlichia* and *Anaplasma* strain has been isolated from wild carnivores around the world so far. Moreover, the isolation of “*Candidatus* Neoerlichia lotoris” in tick cells lines provided enough amount of the pathogen to assess the susceptibility of raccoons and rodents to this new described agent (52). The isolation of new species/genotypes of *Ehrlichia* and *Anaplasma* would also provide substantial amount of antigen, allowing the standardization of serological assays for investigating the exposure of wild and domestic animals and humans to these new agents. In parallel, the isolation of new Anaplasmataceae agents from wild carnivores followed by Whole Genome Sequencing (WGS) will contribute to decrypting the complexity of α-Proteobacteria in wildlife.

The understanding of the epidemiology of canine monocytic ehrlichiosis would benefit from the genotyping of *E. canis* strains found in wild canids and domestic dogs by sequencing the TRP36, a major immunoreactive protein used for investigating the genetic diversity of *E. canis* strains based on differences in tandem repeat number or sequences (115). Would circulate the same TRP-36 genotypes in red foxes (or jackals) and domestic dogs in periurban areas where an overlap of the ecological niches of these canid species is observed? Would there be a predisposition of certain *E. canis* genotypes for species of wild carnivores? *E. canis* genotypes found in humans would be more related to those found in wild or domestic canids? Recently, De Sousa et al. (12) failed to detect the *trp36* sequence in *rrs*-*E. canis* positive blood samples from domestic dogs, crab-eating foxes and coatis from Brazilian Pantanal, which precluded additional genotyping. On the other hand, a new *E. canis*-TRP36 genotype detected in blood donors in Costa Rica showed to be closely related to those detected in Brazilian domestic dogs (69).

Although *E. canis*-like *rrs* and/or *dsb* sequences have been detected in domestic cats from the USA (116), Brazil (117, 118), Portugal (119), and Angola (120), and wild felids from Brazil (75, 9) and Zimbabwe (42), the agent has not been isolated yet, hampering a more deep genetic and antigenic characterization of this agent in felids. The isolation and TRP36-genotyping would shed some light to the genetic diversity of these *E. canis*-like strains, aiming at clarifying if the occurrence of *E. canis* in domestic and wild felids would represent a “spill-over” from infected dogs in endemic areas for canine monocytic ehrlichiosis.

Similarly, the genotyping of *A. phagocytophilum* strains circulating in wild carnivores based on different genetic markers (*rrs, groEL*, and *ankA* genes and 23S-5S rRNA intergenic spacer) (113) will contribute to the assessment of the evolutionary distance among *A. phagocytophilum* clusters formed by strains found in wild carnivores, domestic and wild mammals, ticks and humans, contributing to the understanding of the role of wild carnivores in the epidemiology of human granulocytic anaplasmosis. In this respect, Stephenson et al. (121) designed a qPCR assay targeting the *ank* gene of *A. phagocytophilum*, which showed sensitivity and specificity for the p-Ap genospecies (pathogenic *A. phagocytophilum* strains detected in dogs, horses and humans in the USA), differentiating them from the apparently non-pathogenic DU1 genospecies (found in woodrats [*Neotoma fuscipes*] and bears from California). However, this assay showed cross-reaction with Ap-Variant 1, which is found in deer and goats in the USA. The designed assay will contribute to the screening of wild animals, including carnivores, and vectors in the USA. In areas where an overlap of DU1 and Ap-Variant 1 genospecies is expected, an additional PCR assay to differentiate the latter from p-Aph (122) is needed (121). The “distantly related to human marker” (*drhm*) gene locus has been proposed as a virulence marker for *A. phagocytophilum* isolates. This association was mainly due since this locus was not found in human and dog isolates (123); besides, canine granulocytic anaplasmosis resembles most of the disease aspects of the human anaplasmosis. In western USA, carnivores (dogs, bears and gray foxes) showed both *drhm*-positive and negative strains. On the other hand, virulent strains detected in dogs, humans and horses in eastern USA lacked this locus. Although *drhm* did not seem to indicate host-tropism of *A. phagocytophilum* strains, it may be used as a phylogeographic marker in association with other genes (124).

The expanding universe of Anaplasmataceae agents in wild carnivores and their associate ticks will benefit from the use of next-generation sequencing (NGS). This technology has been used for both detecting tick-borne pathogens and understanding the interactions between pathogenic and non-pathogenic microorganisms (commensals and symbionts) associated to ticks (125) and vertebrate hosts (126). Based on these approaches (which can be performed using several platforms, such as Sanger sequencing of full-length *rrs*, 454-pyrosequencing, Ion torrent, Illumina-sequencing of *rrs* hypervariable regions, etc.), an astonishing diversity of microorganisms has been identified in ticks (125). The main advantage of NGS over PCR-based methods is the use of a detection system that is not biased toward specific microorganisms. Therefore, NGS in association with network analysis will shed some light into the composition of microbial communities associated to ticks and wild carnivores, contributing to the understanding of the role of these hosts in the epidemiology of anaplasmosis, ehrlichiosis and other tick-borne diseases. For instance, Ge et al. (126), when exploring the composition of bacterial community in wild mice and shrews' spleen tissues from Chongming Island, China, found that *Anaplasma, Rickettsia* and *Coxiella* were adjacently clustered by hierarchical analysis. Besides, *Anaplasma*-infection was associated with a specific composition of microorganisms in rodents' spleen tissues.

In this review, molecular and serological evidence of infection by Anaplasmataceae agents was reported in wild carnivores around the world, including in even unexpected areas, such as in the arctic ecosystem (17). While abundant wild carnivores (foxes and golden jackals) may act as reservoirs for these agents, those life-threatened (wolves and wild cats) or presenting limited population size (arctic foxes and Iberian lynxes) and are less prone to play important role in the maintenance of endemic sylvatic life cycles (1). Due to increase in numbers and expansion of geographical ranges, the first group of carnivores, which can be represented by red foxes, may be responsible for the transmission for tick-borne agents to domestic animais and humans, mainly in periurban and urban areas (3).

Since a plethora of *Ehrlichia* and *Anaplasma* agents has been detected in both captive and free-ranging wild carnivores, zoos and conservation institutions should be aware of the circulation of these pathogens in these mammals. Even though free-ranging wild carnivores would likely have a much greater risk of exposure to potentially different pathogens than captive animals, the later are potentially exposed to different pathogens due to proximity to other captive and commensal species that may not be encountered in the wild, despite the use prophylactic measures aiming at avoiding ectoparasites infestation. Therefore, surveillance studies in zoos and safari parks, which comprise important piece in conservation, reproduction and recovery strategies, should be also stimulated. The knowledge of the circulation of Anaplasmatceae agents in wild carnivores should be in the biosecurity list of zoos and conservationist institutions, aiming at achieving their goals of preservation and recovery programs. Special attention should be directed to old or immunologically compromised wild carnivores, mainly those whose population has declined (44). Translocation procedures aiming at establishing new populations or reinforcing existing ones in certain areas, release of captive carnivores into the wild, transference of animals between zoos and maintenance of carnivores in rehabilitation centers can favor the spread of tick-borne *Ehrlichia* and *Anaplasma* agents in non-endemic areas. The real consequence of the introduction of new species/strains into naïve populations is still unknown. Management procedures involving wild carnivores can also predispose to a recrudescence of subclinical Anaplasmataceae infection due to stress-mediated immunosuppression (3).

Despite the considerable progress in the identification of tick-borne Anaplasmataceae agents in wild carnivores, a lot of “gaps” still needs attention in order to draw a clearer picture of the epidemiology of these α-Proteobacteria in wildlife, such as: the real identity of the newly *Anaplasma* and *Ehrlichia* genotypes described in wild carnivores in South America and Africa; the identification of vectors and reservoirs involved in the transmission cycles of “*Candidatus* Neoehrlichia sp.” (FU-98) in Europe and those associated to the new genotypes of Anaplasmataceae agents in South America and Africa; the vectors involved in the transmission cycles of “*Candidatus* Neoehrlichia lotoris” among raccoons in North America; the search for additional transmission routes (transplacental, mechanic, ingestion of infected tissues or ticks, etc.) of these agents among wild carnivores; the confirmation of a certain wild carnivore species as “true” reservoir for a selected Anaplasmataceae agent by carrying out experimental infection followed by xenodiagnosis with known competent vectors; studies aiming at investigating the pathogenicity of new species/*Candidatus* agents; the development of serological assays using the new species/*Candidatus* of *Ehrlichia* and *Anaplasma* antigens in order to perform serosurveillance studies on these agents in humans and wild and domestic animal populations as well as investigating the occurrence of serological cross reactions with other Anaplasmataceae agents; the standardization of MLST (*Multi Locus Sequence Typing*) for *Anaplasma* and *Ehrlichia* aiming at improving the phylogenetic assessment of these agents detected in wild carnivores; an extending use of WGS of *Anaplasma* and *Ehrlichia* strains circulating in wild carnivores; the understanding of the pathobiome associated to the community of Anaplasmataceae agents in both ticks and wild carnivores tissues using NGS.

## Conclusions

- Red foxes are hosts for *Anaplasma* spp. (*A. phagocytophilum, A. ovis, A. platys*), *E. canis* and “*Candidatus* Neoehrlichia sp.” (FU98 strain) and may contribute to the maintaenance of *A. phagocytophilum* in Europe;
- Raccoons are hosts for *E. canis, A. bovis*, “*Candidatus* Neoehrlichia lotoris'e” *A. phagocytophilum*, and play a role in the maintenance of *A. phagocytophilum* in the USA;
- Raccoon dogs may play a role as hosts for *A. bovis* and *A. phagocytophilum*;
- New *Ehrlichia* and/or *Anaplasma* genotypes circulate in wild canids and felids from South America and Africa;
- While *Ehrlichia* sp. closely related to *E. canis* has been reported in wild felids from Brazil and Japan, *Anaplasma* sp. closely related to *A. phagocytophilum* has been detected in wild felids from Brazil and Africa;
- Red foxes and mustelids (otters) are exposed to *E. canis* in countries located in the Mediaterranean basin (Portugal, Spain and Italy), probably as a consequence of spillover from domestic dogs. Similarly, *E. canis* occurs in procyonids in North (raccoons in USA, Spain) and South (coatis in Brazil) Hemispheres, in areas where *E. canis* is frequent in dogs;
- While “*Candidatus* Neoehrlichia lotoris” seems to be a common and specific agent of raccoons in the USA, “*Candidatus* Neoehrlichia sp.” (FU98 strain) seems to show a broader range of hosts, since it has been detected in red fox, golden jackal and badger in Europe so far;
- Brown and black bears seem to play a role as hosts for *A. phagocytophilum* in the North Hemisphere.
- *bovis* has been detected in wild Procyonidae, Canidae and Felidae in Asia and Brazil.

## Author contributions

MA designed the conceptualization and write, review and edited the original draft of the manuscript.

### Conflict of interest statement

The author declares that the research was conducted in the absence of any commercial or financial relationships that could be construed as a potential conflict of interest.
